# Migrations of Green Turtles (*Chelonia mydas*) between Nesting and Foraging Grounds across the Coral Sea

**DOI:** 10.1371/journal.pone.0100083

**Published:** 2014-06-18

**Authors:** Tyffen C. Read, Laurent Wantiez, Jonathan M. Werry, Richard Farman, George Petro, Colin J. Limpus

**Affiliations:** 1 Laboratory of Marine Biology and Ecology, Aquarium des Lagons, Noumea, New Caledonia; 2 EA4243 LIVE, Université de la Nouvelle-Calédonie, Noumea, New Caledonia; 3 Griffith Centre for Coastal Management, Griffith University Gold Coast campus, Queensland, Australia; 4 Ocean and Coast Research, Main Beach, Queensland, Australia; 5 Wan Smolbag Theatre, Port Vila, Vanuatu; 6 Threatened Species Unit, Department of Environment and Heritage Protection, Queensland Government, Queensland, Brisbane, Australia; Deakin University, Australia

## Abstract

Marine megafauna tend to migrate vast distances, often crossing national borders and pose a significant challenge to managers. This challenge is particularly acute in the Pacific, which contains numerous small island nations and thousands of kilometers of continental margins. The green sea turtle, *Chelonia mydas*, is one such megafauna that is endangered in Pacific waters due to the overexploitation of eggs and adults for human consumption. Data from long-term tagging programs in Queensland (Australia) and New Caledonia were analysed to investigate the migrations by *C. mydas* across the Coral Sea between their nesting site and their feeding grounds. A review of data collected over the last 50 years by different projects identified multiple migrations of *C. mydas* to and from New Caledonia (n = 97) and indicate that turtles foraging in New Caledonia nest in the Great Barrier Reef (Australia) and vice versa. Several explanations exist for turtles exhibiting this energetically costly movement pattern from breeding to distant foraging grounds (1200–2680 km away) despite viable foraging habitat being available in the local vicinity. These include hatchling drift, oceanic movements and food abundance predictability. Most of the tag recoveries in New Caledonia belonged to females from the south Great Barrier Reef genetic stock. Some females (n = 2) even showed fidelity to foraging sites located 1200 km away from the nesting site located in New Caledonia. This study also reveals previously unknown migrations pathways of turtles within the Coral Sea.

## Introduction

Human disturbance is triggering unprecedented and mounting biodiversity losses on a global scale, fuelling concerns over species extinctions and the degradation of important habitats [1]. Many charismatic and top-level marine fauna have registered dramatic declines in the last decade [2], [3]. Anthropogenic stressors on marine ecosystems are likely to increase as almost all biodiversity hotspots around the world are expected to at least double their human populations within the next 50 to 100 years [4]. Therefore, the identification of spatial and temporal patterns of abundance, reproduction, demography and capacity for resilience to impacts (including exploitation) is critical for managing the conservation of marine megafauna species including turtles.

The green turtle, *Chelonia mydas*, is a circumglobal species classified as endangered on the International Union for Conservation of Nature (IUCN) Red List due primarily to declines from overexploitation of eggs and adult females at nesting beaches, and juveniles and adults in foraging areas [5]. Additional pressures on *C. mydas* populations come from incidental mortality in marine fisheries and degradation of marine and nesting habitats [5]. Impacts on stocks are exacerbated by the species slow growth, late onset of sexual maturity and low survivorship of hatchlings [6]. Sea turtles are highly migratory and upon reaching sexual maturity utilise broadly separated dispersed neritic foraging grounds and limited localised nesting areas that drive regional distribution patterns [6], [7]. Migrations, often over hundreds of kilometers, are undertaken every few years by both males and females by most sea turtles [8]–[10]. Mature females commonly return from foraging grounds to the region of their natal beach [11]. Species which cover vast distances across international waters, pose a significant challenge for managers. Consequently, identifying migratory paths between nesting and foraging grounds is important for effective transboundary conservation strategies at both the local sub-population level and the regional population level [12]–[14].

In the last decade there has been an exponential increase in innovative tracking technologies enabling identification of the migratory pathways of marine turtles based on a small number of individuals [15]. While these technologies are very useful they lack the ability to identify long-term (over decades) patterns of movement across a large number of individuals. An alternative method is the mark and recapture of individuals using flipper tags [16], [17]. While this method is often intensive it enables the mark and identification of potentially hundreds to thousands of individuals and the identification of large-scale movements if individuals are recaptured at separated nesting beaches or foraging grounds. Furthermore, in conjunction with effort estimates, mark-recapture may enable researchers to derive coarse population estimates [18], [19].

Previous studies have shown long-range migrations by *C. mydas* worldwide [20]–[23], however documented examples from the South Pacific are scant but have to date demonstrated the record movement for this species (3880 km). This was attributed to an individual tagged as an immature female at Clack Reef (Australia) and found nesting 17 years later on Wotje Atoll (Marshall Islands) [23]. While there is sufficient evidence to suggest substantial movements in the southwest Pacific, hypothesised to be driven by site-fidelity [7], most of these examples are based on satellite telemetry or mark-recapture of only a few individuals [20], [23]–[26]. A small number of females have been found to travel from a rookery at Scilly atoll in French Polynesia to multiple distant feeding grounds (<2000 km) in Fiji, New Caledonia, Tonga, Vanuatu and Wallis [24] and from the American Samoa to Fiji [20], [25]. However, between Australia and New Caledonia, numerous tagging campaigns of *C. mydas* have been undertaken with over 80,000 individuals tagged in Australia since 1964, thus providing the potential to identify extensive migratory patterns in the Coral Sea. In this study we used multiple long-term databases on the tagging and recapture of *C. mydas* on the east coast of Australia and in New Caledonia, to (1) determine spatial migratory patterns of tagged *C. mydas* across the Coral Sea to and from New Caledonia, (2) identify temporal patterns of migration, and (3) quantify the patterns of connectivity between foraging and beach nesting areas using both mark-recapture and complimentary examples from satellite telemetry.

## Materials and Methods

### Ethics Statement

This research was executed in accordance with GBRMPA/State Marine Park permit G00/240, and G09/25033.1 and New Caledonian permit 2011-2751/GNC and a Griffith University animal care and ethics approval ENG/01/12/AEC.

### Study Sites

**Figure 1:**
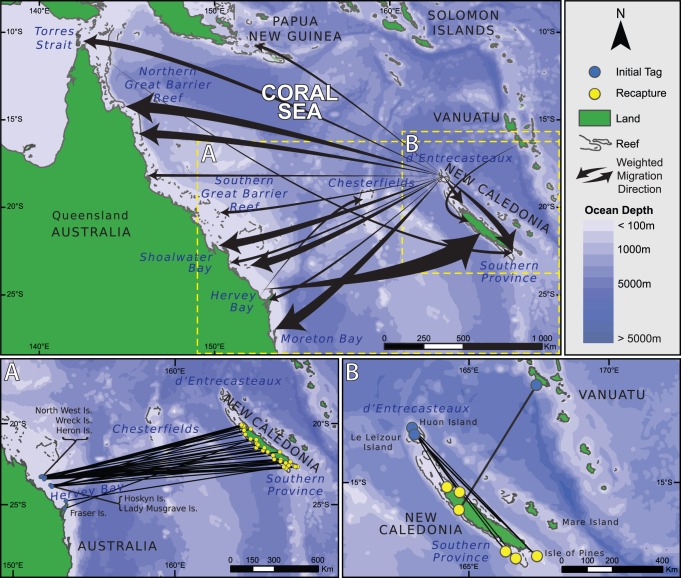
Trajectory maps obtained by the tag recoveries (n = 93) and satellite tracking of *C. mydas* in the Coral Sea (n = 1).

Our study focused on the recapture of tagged *C. mydas* at foraging grounds and nesting beaches between 1972 and 2011 across the spatial extent of the Coral Sea (Figure 1). Australia and New Caledonia border the east and west boundaries of the Coral Sea respectively, while to the north the sea is bordered by the south coast of Eastern New Guinea, the Solomon Islands and Vanuatu.

The study area included four key locations along the east coast of Queensland (QLD), Australia and multiple locations in New Caledonia (NC). These being 1) reef foraging areas within Torres Strait, and the Bramble Cay nesting beach; 2) nesting beaches in the northern GBR (nGBR) including Raine Island, Moulter Cay No.7 and No. 8 Sandbanks and reef and seagrass foraging areas including Clack Island reef and Green Island reef, 3) coral cays of the southern GBR (sGBR) including Heron Island, Northwest Island, Wreck Island, Lady Musgrave Island and Hoskyn Island in the Capricorn-Bunker Groups and Swain Reef’s Cays and associated coral reef foraging areas and coastal pastures in Repulse Bay and Shoalwater Bay, 4) the seagrass pastures of Moreton Bay in southeast QLD; and 5) two nesting locations in the islands north of New Caledonia: D’Entrecasteaux atolls and Chesterfields reefs plus multiple feeding grounds around the main island of New Caledonia.

### Capture and Tagging Efforts

We used recapture data from several long-term tagging programs in QLD and New Caledonia (see acknowledgments for tagging programs). In Australia, these tagging campaigns of *C. mydas* since 1964 have resulted in over 80 000 tagged individuals and over 4000 tagged individuals in New Caledonia. Due to the differences in longevity of different *C. mydas* tagging programs, capture and tagging efforts occurred disproportionally among the study locations as tagging efforts were first initiated in Australia, twenty-seven years prior to tagging efforts in New Caledonia [27]. Furthermore, to provide a more comprehensive understanding of the dynamics and ecology of *C. mydas*, juveniles and males, which are very rarely found ashore, were also tagged as part of this study starting in 1974 [27], [28]. Few migrating *C. mydas* have been tracked via satellite telemetry for their post-breeding migrations within the Coral Sea region. A female was equipped with a satellite tag after nesting at Bamboo Bay, in Vanuatu in 2011 and followed to its feeding area to provide additional information on potential migrations between feeding and nesting grounds in the Coral Sea.

### Tagging

*C. mydas* were captured using different methods depending on their activity in foraging grounds or on nesting beaches. In-water turtles were captured by rodeo method using a small boat or by hand in the shallows [28] and by hand for turtles nesting on land. Prior to tagging, standard measurements of the midline curved carapace length (CCL), and gender, when possible, were recorded. In New Caledonia, all individuals recorded in the database were nesting females tagged on the beach.

Pre-1980, external monel tags with a unique identification number were applied to the anterior fin of captured turtles but due to corrosion tag loss was important. The issue was overcome by using a self-piercing, self-locking titanium identification tag in the front flipper immediately adjacent to the first large scale on the proximal rear edge, close to the axilla [17]. Probability of loss after 9 years of the tag being applied was reduced from 1 to 0.667 (±0.533) by switching from Monel No. 49 tags to titanium tags No.2 [17]. The position at which the tags was applied was also tested and position 3 (closer to the axilla) decreases the probability of loss compared to position 1 (at the tip of the front flipper) and position 2 (in the middle of the front flipper) [17]. Double tagging was implemented as some tag loss occurred during agonistic interactions at courting but also due to the environment (probably due to digging, encountered rocks and branches, plus crawling) [17], [29], [30]. Satellite telemetry was also used on a single individual in Vanuatu to test the potential connectivity with other countries within the Coral Sea and to explore future titanium tagging sites.

### Movements

The movements of tagged *C. mydas* between individual locations were recorded through the reported recaptures of individuals either within ongoing tagging efforts in Queensland, New Caledonia and other regions in the south-west Pacific or via fishermen and local people. Identification tags enabled the verification of individual movements both spatially and temporally.

### Statistical Analyses

All analyses were completed using Statgraphics. Significance was determined as 0.05. The minimum linear distance between tag and recapture locations was determined using Google Earth and used to identify the extent of movement by individual turtles within the Coral Sea population. Homoscedasticity of tag recoveries (having equal variance) was verified using Bartlett’s test and the mean difference in curved carapace length (CCL) between Australia and New Caledonia was compared using a t-test. The nesting beach release point and the feeding ground of the satellite tagged turtle in Vanuatu were used as single capture and recapture locations.

## Results

The major breeding aggregations of *C. mydas* in the south western Pacific region are known to represent independent genetic stocks or management units [12], [31]. In Australia, seven different breeding stocks have been identified to this day: southern Great Barrier Reef (sGBR), Coral Sea, northern GBR (nGBR), Gulf of Carpentaria, Ashmore Reefs, Scott Reef and the Northwest Shelf [12], [31]–[34]. Based on tag recovery data analysed in the present study, *C. mydas* foraging within New Caledonian waters originate from at least four independent genetic stocks breeding in at least four different countries: New Caledonian stock (n = 49); Australian stocks (sGBR (n = 45), nGBR (n = 2) stocks) and probably an independent stock in Vanuatu (n = 1). No individuals from the Australian Coral Sea stock have been identified foraging in New Caledonia.

*C. mydas* tagged while nesting in New Caledonia have been recaptured as foraging turtles in three countries: New Caledonia, Australia and Papua New Guinea. A total of 4700 individuals were tagged at D’Entrecasteaux atolls (New Caledonia), resulted in only a 1% post-nesting migration tag recovery. Females (n = 46) nesting at D’Entrecasteaux atolls were found in feeding grounds all along the Queensland coast (n = 37) up to Papua New Guinea (n = 1) but also in New Caledonian waters (n = 8) (Figure 1). One female tagged in Australia was reported nesting the same year on an island of the Chesterfield atolls (Figure 1).

Less than 0.1% of the *C. mydas* tagged in Australia were recovered in New Caledonian foraging area. Females (n = 45) tagged at nesting beaches in the Great Barrier Reef were found in feeding grounds in New Caledonia illustrating reciprocal movements across the Cora Sea in both a westerly and easterly direction (Figure 1). One female (“Bamboo Lady”) was equipped with a satellite tag in Vanuatu while nesting and came to New Caledonia to forage (Figure 1).

**Figure 2:**
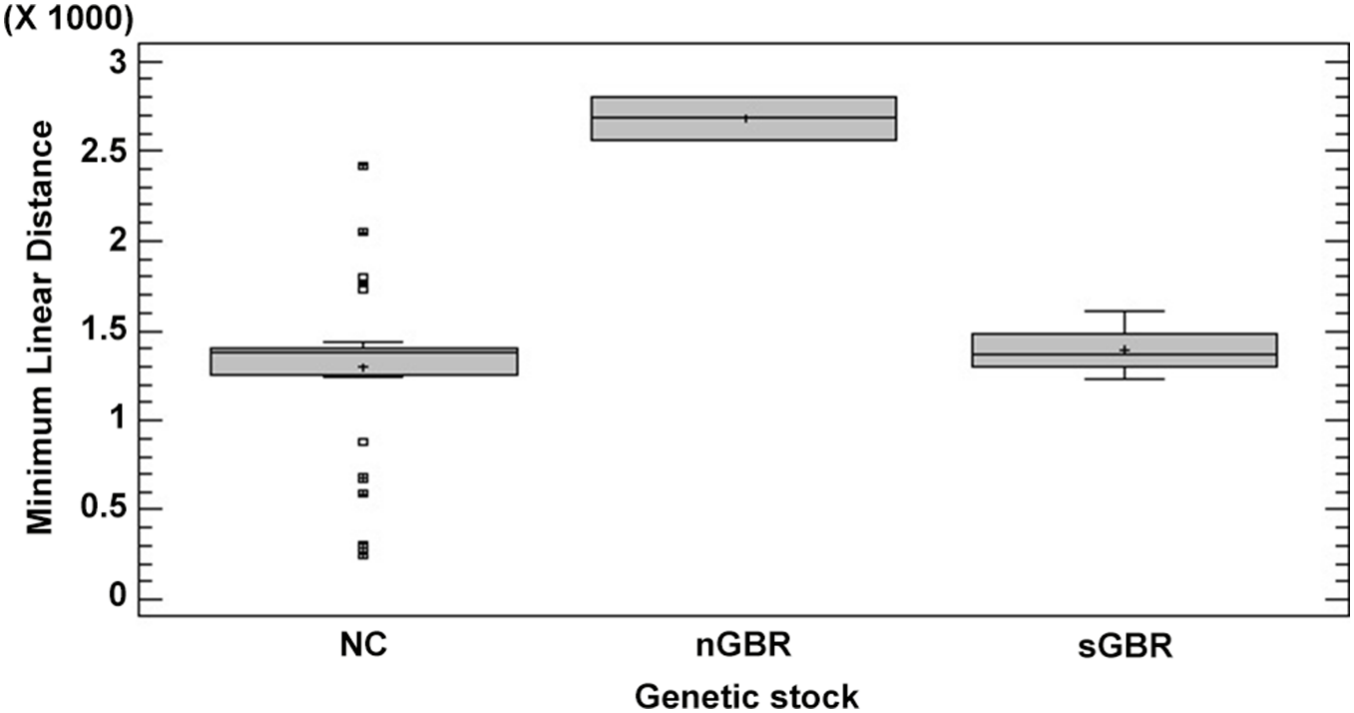
Mean linear minimum distance (km) (± SE) from initial tagging locations in the Coral Sea. nGBR (n = 2), sGBR (n = 39) and NC (n = 45).

**Figure 3:**
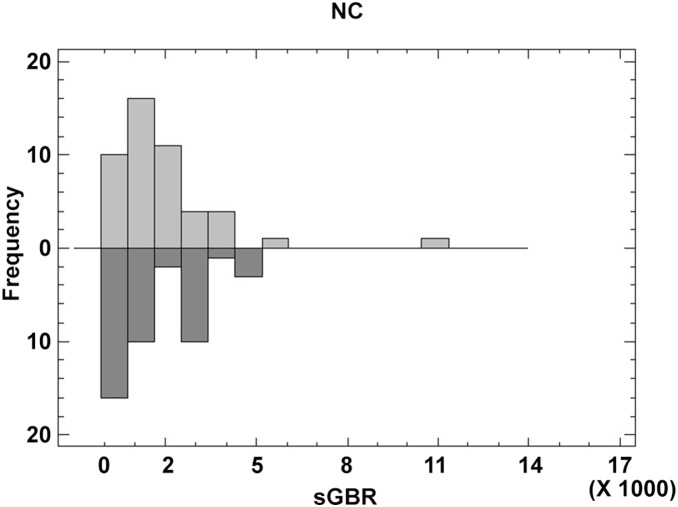
Mean time (days) from initial tagging locations in the Coral Sea. sGBR (n = 31) and NC (n = 58).

**Figure 4:**
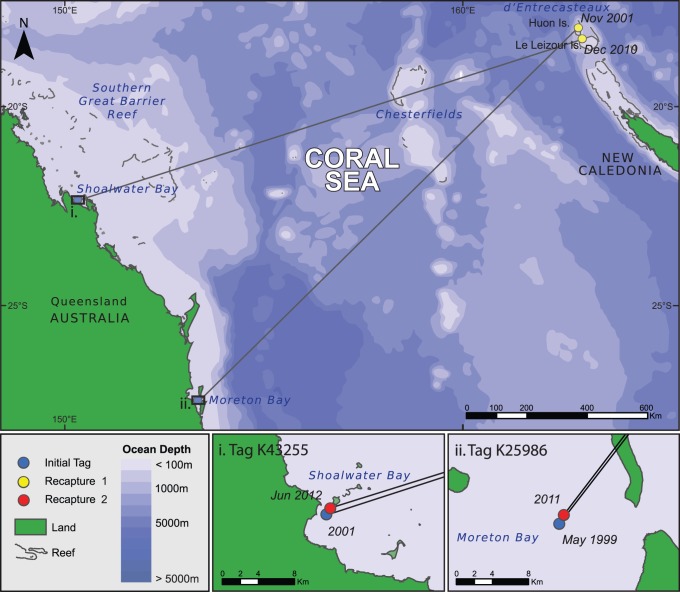
Foraging site fidelity recorded by two females *C. mydas* originally tagged in Australia, found nesting in New Caledonia and recaptured in subsequent years back at their respective feeding grounds.

Distances traveled between foraging and nesting grounds were significantly different (Krustal-Wallis test, p = 0.03), the longest being between NC and nGBR (2680 km) (Figure 2). Here we made an assumption that all individuals were caught in their feeding ground as they were either in a known feeding ground or hunted in coastal waters by local tribes. The mean time for tag recoveries was not significantly different between females belonging to NC and sGBR genetic stocks (F test, p>0.15) (Figure 3). The minimum time for a tag recovery in this study was 19 days and the maximum was 10585 days with a mean of 1756±162 days. Means were not able to be determined for nGBR and Vanuatu due to low number of recaptures (respectively n = 2 and n = 1). However, satellite telemetry revealed it took 12 days for the turtle to travel from Vanuatu to its feeding area and the tags from the only two females from nGBR were recovered in New Caledonia 584 and 1265 days after initial tagging. Two females tagged at their feeding grounds in Australia, reported nesting in New Caledonia were recaptured at their initial capture site (Table 1). Both females from the D’Entrecasteaux rookery, however, were recaptured at separate locations in Australia. One forages in Moreton Bay while the second occurred in Shoalwater Bay (Figure 4).

**Table 1:** Case histories illustrating fidelity of *C. mydas* to feeding areas in Coral Sea.

Tag No.	Behaviour	History
K 25986	Feeding	Moreton Bay in 1999, sub-pubescent CCL: 102
	Nesting	Huon Island (NC) in 2001, first breeding season
	Feeding	Moreton Bay in 2011 CCL: 106.1
K43255	Feeding	SWB sub-pubescent 2001 CCL: 88.1
	Nesting	Fabre Island (NC) in 2010
	Feeding	SWB Jun2012 non breeding adult CCL: 93.1

**Figure 5:**
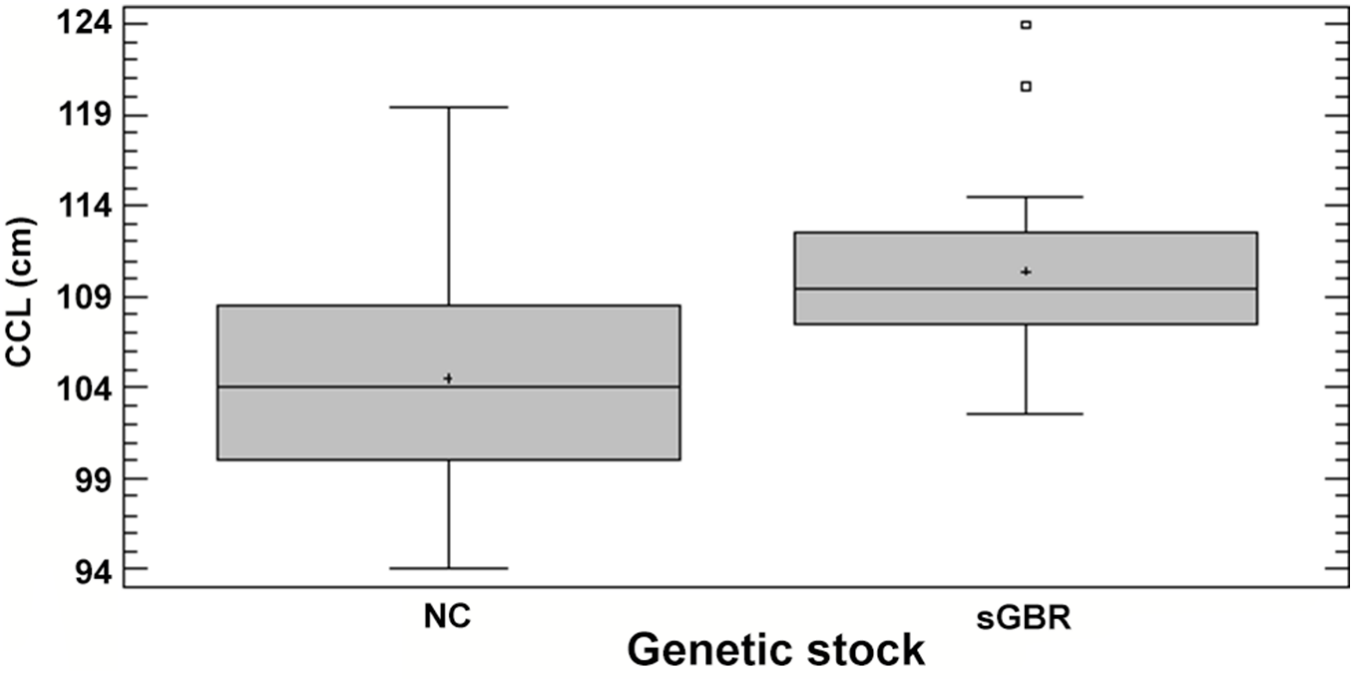
Mean CCL (cm) (± SE) at two nesting areas in the Coral Sea. sGBR (n = 15) and NC (n = 32).

**Figure 6:**
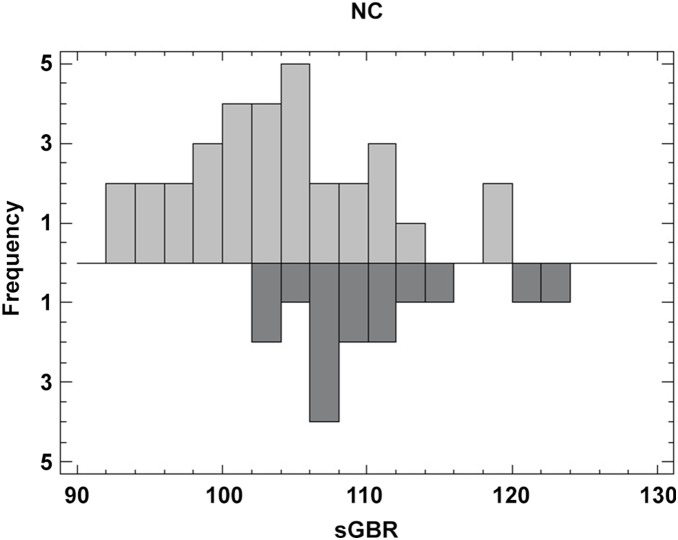
Distribution of the mean CCL (cm) at two nesting areas in the Coral Sea. sGBR (n = 15) and NC (n = 32).

Females were either tagged during a nesting event or recovered at their nesting site and their CCL was recorded during this time (Figure 5). The mean CCL for females recorded in this study nesting at D’Entrecasteaux atolls is 104.5 cm (SE±2.4) compared to a mean CCL of 110.4 cm (SE±3.3) for females nesting in Australia (recorded in this study). No significant difference was found between the CCL of females recorded in this study nesting at D’Entrecasteaux atolls and the CCL of all females reported nesting at D’Entrecasteaux (t-test, p = 0.7). A significant difference was found between the size of individuals nesting in NC and individuals nesting in sGBR, and known to travel across the Coral Sea (we excluded individuals that were known to nest in NC and recaptured at their feeding site in NC, n = 6) (t-test, p = 0.00). The individual from Vanuatu was removed from the analysis (which had a CCL of 102 cm) and the two individuals from nGBR were also excluded (did not have their CCL recorded). The distribution of the size recorded from each female at its nesting site is uneven due to a paucity of female size data (Figure 6).Six percent of the individuals tagged in Australia and found in New Caledonia were males (n = 4). They were excluded from all analysis due to their low numbers but table 2 recapitulates the data that was collected for these individuals.

**Table 2:** Case histories illustrating male *C. mydas* migration across the Coral Sea.

PTAG	TAG	CCL (cm)	PLACE	NPLACE	EDAYS	MinDistance (km)
T	15584		NC:PUETEGE RF,Merlet	sGBR:CBG:HERON RF	3795	1580
T	78838		NC:BALABIO	sGBR:HERON ISLAND	4410	1335
X	22681	98,0	NC:MIDWEST	sGBR:HERON ISLAND	580	
T	78636		MB:MORETON RODEO	NC:ILE HUON-ON BEACH	4552	1419

## Discussion

Long-term databases on the tagging and recapture of *C. mydas* on the east coast of Australia and in New Caledonia revealed multiple migrations across the Coral Sea showing heterogeneous patterns in connectivity between *C. mydas* nesting and feeding grounds across the Coral Sea. Despite low tag recovery, migratory paths spanned the entirety of the Coral Sea with considerable longevity between mark and recaptures periods.

### Low Tag Recovery

Low tag recoveries could be explained by several factors. Firstly, a very high dispersal rate in the South Pacific. The individuals found nesting in New Caledonia are likely to be foraging on every reef and seagrass pasture within the Southwest Pacific region, thus making it difficult to recapture them (or be informed of their recapture) from areas with low human populations. Secondly, very high loss rate of tags. If a turtle loses its tag then when it is encountered again it is not seen as a recapture. However, there are much higher rates of tag recoveries at dedicated tagging–recapture study sites in Australia of breeding *C. mydas* returning for nesting in subsequent years [35] and in foraging areas [36], [37]. Thirdly, high anthropogenic impact. The low proportion of recaptures could be explained by high numbers being killed for food consumption, as bycatch or by pollution; and finally low percentage of tag return. People are often reluctant to return tags to the appropriate authorities as it is prohibited to hunt turtles in New Caledonia since 2009 (although exceptions are made for traditional purposes).

### Post–Nesting Trends

The different species of sea turtles are dissimilar in many ways, but one behavior they have in common is that they return to the area where they hatched in order to reproduce, a phase often referred to as “natal homing” [38]. A way to study this period is to tag turtles as they are leaving the beach after nesting and study their migration back to their feeding grounds. Many studies have looked at different hypothesis on how certain taxa migrate [39]–[41] and many specifically on sea turtles [42]–[44] but very little is known on why and what pathways do they use. Here we try to provide records to understand the underlying patterns of *C. mydas* dispersion in the South Pacific by looking at the tag recoveries geographically. Tag recoveries from females tagged at D’Entrecasteaux atolls were found all along the QLD coast, in PNG and also around the main island of New Caledonia. We found a very clear west-ward trend for post-nesting migration when nesting occurred in New Caledonia. This has been reported previously from the central South Pacific where 96% of individuals in the study migrated westward and more specifically to Fiji [45]. The explanation given was that Fiji has large areas of seagrass and algae. No record has been found of *C. mydas* migrating from New Caledonia to Fiji but two individuals have also been recorded to travel from French Polynesia to New Caledonia, thus going past Fiji [24]. Within the same study, five individuals were tracked from French Polynesia to Fiji [24]. More factors are likely at play to explain the observed westward trend besides the abundance of food resources. Moreover, three females known to nest in New Caledonia were found feeding on Heron Reef, while 23 females known to nest on Heron Island were found in New Caledonia. Turtles born on Heron Island therefore seem to have enough forage near their nesting grounds so why travel 1300 km to feed, with increased energy demands associated with long migration. Taken together, these findings lend strength to the hypothesis that more cues are used by sea turtles to choose their feeding grounds than just the abundance and proximity of food sources. Several species from the family Salmonidae have a similar pattern of natal-homing [46], [47] but their migration seems to be explained by a feeding pattern and a trail of pheromones left by descending smolt that triggers the migration [48], which is not proven in sea turtles. The composition of foraging aggregations seems to be also influenced by currents and the Earth’s geomagnetic field [49], [50] but not all individuals choose to settle in the exact same way, otherwise individuals coming from the same rookery and born in the same year at one rookery would all be found in one feeding ground, which is not the case [23], [37], [51]. A recent hypothesis is that foraging site selection reflects passive drift experienced by hatchlings thus the adult’s movements seems to be directed by constant currents from breeding sites [52]. The North Caledonian Jet (NJC) and the South Caledonian Jet (SCJ) both have a western direction thus possibly pushing hatchlings towards Australia. However, this does not explain the eastern trend of turtles known for nesting in Australia and found feeding in New Caledonian waters as these currents now act as restraints [53]. This pattern of ocean crossing is also found in loggerhead turtles (*Caretta caretta*). This was proven genetically and by tag recoveries, showing individuals feeding in Australia and belonging to multiple rookeries in the South Pacific (including New Caledonia) [54], [55]. Tag recoveries have also showed *C. caretta* foraging in feeding grounds spread in the Pacific Ocean and nesting in Australia [7]. Once again, it is indicated that the migration is not due to a lack of resources but rather an intricate pattern during the “lost years”. Together these results indicate that other significant factors are yet to be identified in order to fully understand the components of recruitment and migration patterns in sea turtles and more specifically for *C. mydas*.

### Stocks

Females that come to nest at D’Entrecasteaux atolls, the nGBR nesting beaches and the sGBR nesting beaches are from independent genetic stocks [56], [57]. There are many sea turtles studies that demonstrate that the turtles nesting at one beach migrate from numerous widely dispersed foraging sites and that turtles living in any one foraging area will have originated from multiple genetic stocks [33], [42], [56], [57]. However, based on available tag recoveries, the foraging *C. mydas* population of New Caledonia is dominated by turtles from the sGBR stock. Seventeen percent of post-nesting migration tag recoveries from *C. mydas* tagged while nesting in New Caledonia have been recorded from New Caledonian waters and ninety-five percent of recaptured foraging green turtles in New Caledonia that came from Australian nesting beaches come from the sGBR. Only two individuals from the nGBR were recovered in New Caledonia. Knowing that the nGBR and the sGBR populations are genetically distinct, this data shows that the resident populations of *C. mydas* in New Caledonia have a higher percentage of individuals belonging to the sGBR than the nGBR genetic group. This correlates with the data collected in Australia, where the frequency of tag recoveries originating from the sGBR genetic stock increases along eastern Australia south from Torres Strait (9°S) to central New South Wales (33°S) [28]. In the results it was indicated that *C. mydas* foraging within New Caledonian waters originate from at least four independent genetic stocks breeding in at least four different countries: New Caledonian stock (n = 47); Australian stocks (sGBR (n = 45), nGBR (n = 2) stocks) and probably an independent stock in Vanuatu (n = 1). Mitochondrial DNA from females nesting at Chesterfield atolls has not been tested yet. It is highly probable that it will add a second independent stock in New Caledonia as the two rookeries are separated by more than 500 km [57].

It was noted that no individuals from the Australian Coral Sea stock [12], [27] have been identified foraging in New Caledonia. This should be investigated further along with the data originating from French Polynesia [24]. Two individuals were tagged while nesting at Scilly Island in French Polynesia only to be recovered foraging in New Caledonia. This data adds a fifth independent genetic stock found in *C. mydas* foraging in New Caledonia and indicates that the genetic diversity of the sea turtle population in the Coral Sea is yet to be fully understood.

### Distance Travelled and Timing between Mark and Recapture

The low number of individuals originating from the nGBR can be explained by the distance that has to be travelled (>2000 km) and the energetic cost that they incur [58]. Many studies report post-nesting migrations of *C. mydas* in the range of 10′s of km to 1500 km in [3], [7], [23], [43], [51] but recorded migrations over 2000 km are also part of the ecology of this species in the Pacific [7], [22], [26], [28], [37], [44], [56], [59]–[64]. Our findings are broadly in accord with the global patterns of migration distances for adult Cheloniid turtles and “similar to that predicted for equivalent-sized marine mammals and fish” [65].

Mean time between initial tagging and tag recovery is not significantly different for the individuals that belong to the New Caledonian and sGBR genetic stock. This can be explained by the fact that a large proportion of tag recoveries from both countries come from hunters and members of the public who report stranded turtles. Here we are reporting on how long it takes to recover a tag not how long it takes a turtles to travel between its foraging and breeding grounds. More field work is needed in New Caledonia and other South Pacific Islands to narrow the mean time of recapture between foraging and nesting grounds to calculate precisely the time frame needed for these individuals to cover those distances and look at the interval between nesting at D’Entrecasteaux atolls. The time for tag recovery in New Caledonia can partly be explained by the lack of an organised program that is necessary to reach remote tribes and educate local populations on the purpose of those tags. All tag recoveries from individuals found feeding in New Caledonia and tagged in Australia were done by fishermen that hunted these turtles for food. It is highly probable that many more females undertake that migration, yet tags are not returned and the data therefore does not reflect the true dynamics of *C. mydas* in the Coral Sea.

### Feeding Site Fidelity

From the individuals known to nest in New Caledonia, two were recaptured at a later date back in their original tagging area at their feeding site in Australia. As showed in the results, the first individual (K25986) was originally tagged in Moreton Bay (MB) in 1999 and recaptured in that same Bay in 2011. The second individual (K43255) was caught at Shoalwater Bay (SWB) in 2001 and recaptured at the same location in 2012. This shows some fidelity of *C. mydas* females to their foraging grounds, even though their nesting site and their foraging site are separated by 1200 km. This behavior has been recorded elsewhere but with shorter distances (in the order of ten to hundreds of km) [7], [66].

### Size

The significant difference found in CCL between females originating from foraging grounds in Australia versus adults caught on New Caledonian feeding grounds is in concordance with other studies. *C. mydas* living in different locations have different sizes [28], [37], [67]. These differences can be explained by foraging-ground-dependent growth rates [67]. Because *C. mydas* may have a high fidelity to their foraging site (as shown above), their size could reflect the quality of their feeding grounds. The mean CCL for females used in this study recorded nesting at D’Entrecasteaux atolls is not significantly different to the mean of all recorded females nesting on these atolls [68]. In this study, the mean size for females at their nesting grounds in the SGBR is 110.4 cm compared to the historical data giving a mean CCL of 107.0 cm for females nesting at Heron Island (representative of the sGBR) [69]. Studies are needed in New Caledonia in order to calculate growth rates at feeding sites.

### Male Turtles

Out of all of the tags recovered in New Caledonia and belonging to individuals originally tagged in Australia, only 6% (n = 4) belonged to males. At SWB, the mean sex ratio (female: male) for adults caught is 1∶1.78 [37] compared to 1∶0.80 if we look at 4 different feeding grounds within the GBR [69]. As sea turtles have temperature-dependent sex determination (TSD), skewed sex ratios in a population over a period over time can lead to the disappearance of a population [70]. It is known that males also migrate to the area where they were born in order to reproduce [71], [72] and a study reported that the breeding periodicity for male sea turtles is 2.6 times more often than females [70]. Three of the males in the database were caught by fisherman in their NC foraging area, and one of the males was encountered on the beach at Huon Island (D’Entrecasteaux atolls) during nesting season. This individual appeared to have been basking [73]. Tagging programs typically focus on females as large number of individuals can easily be tagged on the beach during nesting season. However, tagging males (or juveniles) is important if we are to fully understand population dynamics in *C. mydas* and devise effective management and conservation programs. This is all the more important as understanding the fate of juvenile life history stages is an important determinant of population changes in sea turtles [74].

### New Trajectories

As reported in the results, two migrations paths were unraveled within this study. The first ever recorded turtle migration between Australia and the Chesterfield atolls (New Caledonia). QA 14889 was originally tagged by the Queensland Turtle Research program in western Harvey Bay (Booral) in Queensland on the 30^th^ of April 2011. It was seen nesting on Bampton Island (Chesterfield atoll) on the 19^th^ of November 2011. It has been reported that the Chesterfield atolls are an area of importance for sub-adults and adult male tiger sharks that move between the GBR and New Caledonia [75]. More research in that area is needed in order to test the hypothesis that these reciprocal movements of this top level predator may reflect on *C. mydas* migrations. Secondly, the post-nesting migration of a female “Bamboo Lady” from Bamboo Bay in Vanuatu to its feeding ground in Voh (New Caledonia) is the first recorded migration of sea turtle between those two countries. New trajectories of megafauna in the Coral Sea are being recorded now that these secluded areas are starting to being investigated.

## Conclusion

Most of the tags recovered from *C. mydas* individuals in New Caledonia belonged to turtles known to nest in the sGBR of Australia. New migrations paths were uncovered for *C. mydas* in the Pacific region between the Chesterfield atolls (in New Caledonia) to Australia and Vanuatu to New Caledonia. This study reinforces that *C. mydas* travel long distances (>2000 km) between their feeding and nesting grounds in the Coral Sea. The low percentage of tag recoveries, however, needs to be better explained. Is this just due to a lack of tag returns or do the numbers of recaptures reflect the actual importance of migrations throughout the Coral Sea (i.e., with low tag recoveries explained by the lack of capacity at the regional scale)? Findings reported here demonstrate the need for a comprehensive tag recovery program in New Caledonia. Most of all, this study confirms that sea turtle conservation is not a localised management problem, but rather an international issue and management activities need to be devised and implemented at a larger scale: in this instance across the Coral Sea.
